# Supplementary material for: Proteomic Analysis of Zn Depletion/Repletion in the Hormone-Secreting Thyroid Follicular Cell Line FRTL-5
Source: Nutrients. 2018 Dec 14;10(12):1981. doi: 10.3390/nu10121981 (PMC6315927; doi:10.3390/nu10121981)
Supplement: Supplementary file 1 [file nutrients-10-01981-s001.zip › Table S1.docx]

**Table S1:** log2 fold change (FC) of proteins modulated according to mass spectrometry analysis. Proteins that don’t reach the minimum log2 FC threshold of +/-1 are considered not modulated

| **PROTEIN ID** | **RECvsTPEN** | **RECvsCTRL** | **TPENvsCTRL** | **MODULATED**  **REC vs TPEN** | **MODULATED**  **REC vs CTRL** | **MODULATED**  **TPEN vs CTRL** | **CLUSTER** |
| --- | --- | --- | --- | --- | --- | --- | --- |
| **SHPK** | -3,91 | -2,11 | 1,80 | D | D | U | **TPEN vs CTRL / RECOVERY vs TPEN** |
| **RPL14** | -2,57 | -1,94 | 0,63 | D | D |  |  |
| **RPS7** | -2,24 | -1,26 | 0,99 | D | D |  |  |
| **WBP11** | -1,93 | -1,21 | 0,25 | D | D |  |  |
| **DPP8** | -1,41 | -1,08 | 0,31 | D | D |  |  |
| **PRDM16** | -1,41 | -1,08 | 0,31 | D | D |  |  |
| **NPIP** | -1,32 | -1,32 | -0,51 | D | D |  |  |
| **HIVEP1** | -1,28 | -1,2 | 0,08 | D | D |  |  |
| **GNA11** | -1,25 | -1,15 | 0,07 | D | D |  |  |
| **RCC1** | -1,19 | -1,75 | 0,23 | D | D |  |  |
| **PTK2B** | -1,15 | -1,07 | 0,53 | D | D |  |  |
| **VDAC2** | -1,12 | -1,21 | -0,51 | D | D |  |  |
| **RAB8B** | -1,10 | -1,22 | 0,02 | D | D |  |  |
| **APOB** | -3,04 | 1,00 | 4,71 | D | U | U | **TPEN vs CTRL / RECOVERY vs TPEN** |
| **CACNA1E** | -2,55 | -0,86 | 1,70 | D |  | U | **TPEN vs CTRL / RECOVERY vs TPEN** |
| **CNN3** | -2,56 | 0,54 | 3,35 | D |  | U | **TPEN vs CTRL / RECOVERY vs TPEN** |
| **ARHGAP20** | -2,51 | 0,04 | 1,55 | D |  | U | **TPEN vs CTRL / RECOVERY vs TPEN** |
| **B4GALT5** | -2,24 | -0,72 | 1,26 | D |  | U | **TPEN vs CTRL / RECOVERY vs TPEN** |
| **RPS5** | -2,04 | -0,79 | 1,58 | D |  | U | **TPEN vs CTRL / RECOVERY vs TPEN** |
| **P41161-2** | -1,83 | 0,09 | 2,20 | D |  | U | **TPEN vs CTRL / RECOVERY vs TPEN** |
| **RPL15** | -1,62 | -0,14 | 1,48 | D |  | U | **TPEN vs CTRL / RECOVERY vs TPEN** |
| **RAN** | -1,52 | -0,16 | 1,13 | D |  | U | **TPEN vs CTRL / RECOVERY vs TPEN** |
| **MAML1** | -1,34 | -0,69 | 1,22 | D |  | U | **TPEN vs CTRL / RECOVERY vs TPEN** |
| **RPL27A** | -1,21 | 0,17 | 1,15 | D |  | U | **TPEN vs CTRL / RECOVERY vs TPEN** |
| **ERP29** | -1,17 | 0,15 | 1,32 | D |  | U | **TPEN vs CTRL / RECOVERY vs TPEN** |
| **AKR1B15** | -1,08 | 0,70 | 2,40 | D |  | U | **TPEN vs CTRL / RECOVERY vs TPEN** |
| **NACA** | -1,05 | 0,56 | 1,61 | D |  | U | **TPEN vs CTRL / RECOVERY vs TPEN** |
| **RASGRP1** | -1,01 | -0,01 | 1,14 | D |  | U | **TPEN vs CTRL / RECOVERY vs TPEN** |
| **TPI1** | -1,00 | -0,18 | 1,00 | D |  | U | **TPEN vs CTRL / RECOVERY vs TPEN** |
| **ZC2HC1A** | -3,80 | 0,00 | 0,00 | D |  |  | **RECOVERY vs TPEN** |
| **IL12RB1** | -3,46 | 0,00 | 0,00 | D |  |  | **RECOVERY vs TPEN** |
| **GNB1** | -2,08 | 0,00 | 0,00 | D |  |  | **RECOVERY vs TPEN** |
| **EIF5B** | -1,88 | 0,00 | 0,00 | D |  |  | **RECOVERY vs TPEN** |
| **SPTBN1** | -1,50 | -0,96 | 0,47 | D |  |  | **RECOVERY vs TPEN** |
| **RPL5** | -1,52 | -0,58 | 0,26 | D |  |  | **RECOVERY vs TPEN** |
| **MICB** | -1,49 | -0,65 | 0,65 | D |  |  | **RECOVERY vs TPEN** |
| **SASH1** | -1,43 | -0,88 | 0,65 | D |  |  | **RECOVERY vs TPEN** |
| **MICU3** | -1,17 | -0,53 | 0,28 | D |  |  | **RECOVERY vs TPEN** |
| **FAM149B1** | -1,13 | -0,51 | 0,45 | D |  |  | **RECOVERY vs TPEN** |
| **ATP5O** | -1,13 | -0,38 | 0,40 | D |  |  | **RECOVERY vs TPEN** |
| **SSPO** | -1,13 | -0,28 | 0,51 | D |  |  | **RECOVERY vs TPEN** |
| **PRDX3** | -1,12 | 0,00 | 0,00 | D |  |  | **RECOVERY vs TPEN** |
| **U3KPS6** | -1,06 | -0,45 | 0,96 | D |  |  | **RECOVERY vs TPEN** |
| **SFI1** | -1,07 | -0,13 | 0,91 | D |  |  | **RECOVERY vs TPEN** |
| **VCL** | -1,04 | -0,17 | 0,87 | D |  |  | **RECOVERY vs TPEN** |
| **ABCC10** | -1,02 | -0,13 | 0,63 | D |  |  | **RECOVERY vs TPEN** |
| **RPL12** | -1,01 | -0,88 | 0,17 | D |  |  | **RECOVERY vs TPEN** |
| **ANKRD30A** | 2,08 | 1,20 | -1,28 | U | U | D | **TPEN vs CTRL / RECOVERY vs TPEN** |
| **SHMT2** | 2,43 | 1,42 | -1,50 | U | U | D | **TPEN vs CTRL / RECOVERY vs TPEN** |
| **CREBL2** | 1,22 | 1,69 | 0,37 | U | U |  |  |
| **RAB6B** | 1,47 | 1,19 | -0,87 | U | U |  |  |
| **AFAP1L2** | 1,76 | 1,31 | -0,63 | U | U |  |  |
| **FAM71E2** | 1,81 | 1,28 | -0,53 | U | U |  |  |
| **PPP2R3B** | 2,63 | 3,77 | -0,85 | U | U |  |  |
| **AHNAK** | 1,66 | -0,35 | -1,04 | U |  | D | **TPEN vs CTRL / RECOVERY vs TPEN** |
| **ENO2** | 1,87 | 0,42 | -1,73 | U |  | D | **TPEN vs CTRL / RECOVERY vs TPEN** |
| **SSB** | 2,01 | 0,44 | -1,81 | U |  | D | **TPEN vs CTRL / RECOVERY vs TPEN** |
| **CNGB3** | 2,25 | 0,48 | -1,85 | U |  | D | **TPEN vs CTRL / RECOVERY vs TPEN** |
| **ST13** | 2,69 | 0,32 | -2,38 | U |  | D | **TPEN vs CTRL / RECOVERY vs TPEN** |
| **RAP1B** | 1,02 | 0,70 | -0,31 | U |  |  | **RECOVERY vs TPEN** |
| **VOPP1** | 1,25 | 0,38 | -0,64 | U |  |  | **RECOVERY vs TPEN** |
| **HSP90B2P** | 1,32 | 0,28 | 0,24 | U |  |  | **RECOVERY vs TPEN** |
| **TTN** | 1,53 | 0,66 | -0,91 | U |  |  | **RECOVERY vs TPEN** |
| **SFPQ** | 1,86 | 0,12 | 0,63 | U |  |  | **RECOVERY vs TPEN** |
| **PKN1** | 0,29 | -3,28 | -3,58 |  | D | D |  |
| **SRL** | -0,88 | -2,39 | -1,51 |  | D | D |  |
| **CALM1** | -0,16 | -1,56 | -1,40 |  | D | D |  |
| **HNRNPA1** | -0,95 | -2,00 | -1,05 |  | D | D |  |
| **PSMD11** | -0,23 | -1,10 | -1,02 |  | D | D |  |
| **SLAIN1** | -0,91 | -1,53 | -0,55 |  | D |  | **RECOVERY vs CTRL** |
| **RCOR3** | -0,22 | -1,38 | -0,75 |  | D |  | **RECOVERY vs CTRL** |
| **YWHAZ** | -0,75 | -1,34 | -0,59 |  | D |  | **RECOVERY vs CTRL** |
| **ACTG2** | -0,47 | -1,22 | 0,81 |  | D |  | **RECOVERY vs CTRL** |
| **ZNF277** | -0,78 | -1,20 | -0,38 |  | D |  | **RECOVERY vs CTRL** |
| **ACLY** | -0,20 | -1,15 | -0,14 |  | D |  | **RECOVERY vs CTRL** |
| **KIF21B** | -0,86 | -1,09 | -0,10 |  | D |  | **RECOVERY vs CTRL** |
| **TECPR1** | -0,90 | -1,04 | 0,14 |  | D |  | **RECOVERY vs CTRL** |
| **F6XSS0** | 0,64 | 1,84 | 1,00 |  | U | U |  |
| **DMD** | 0,81 | 1,97 | 1,22 |  | U | U |  |
| **SKIL** | -0,86 | 1,54 | 2,40 |  | U | U |  |
| **GLUD2** | 0,69 | 6,31 | 4,97 |  | U | U |  |
| **RPL38** | 0,42 | 1,07 | 0,65 |  | U |  | **RECOVERY vs CTRL** |
| **SYNCRIP** | 0,55 | 1,12 | 0,48 |  | U |  | **RECOVERY vs CTRL** |
| **HSPE1** | 0,93 | 1,13 | 0,31 |  | U |  | **RECOVERY vs CTRL** |
| **APRT** | 0,89 | 1,17 | -0,21 |  | U |  | **RECOVERY vs CTRL** |
| **CALR** | 0,34 | 1,24 | 0,64 |  | U |  | **RECOVERY vs CTRL** |
| **MCM6** | 0,03 | 1,73 | 0,46 |  | U |  | **RECOVERY vs CTRL** |
| **HIST1H2AJ** | 0,64 | 2,16 | 0,35 |  | U |  | **RECOVERY vs CTRL** |
| **SEPT3** | 0,78 | -0,65 | -1,39 |  |  | D |  |
| **PTCHD1** | 0,69 | -0,29 | -1,32 |  |  | D |  |
| **NEK5** | -0,13 | -0,39 | -1,17 |  |  | D |  |
| **RPL9** | -0,62 | -0,96 | -1,16 |  |  | D |  |
| **FSTL3** | -0,72 | 0,28 | 1,00 |  |  | U |  |
| **TALDO1** | -0,91 | 0,36 | 1,00 |  |  | U |  |
| **ANXA5** | -0,18 | 0,94 | 0,99 |  |  | U |  |
| **PPP1CB** | -0,85 | 0,08 | 1,04 |  |  | U |  |
| **MDH1** | -0,83 | 0,08 | 1,12 |  |  | U |  |
| **PYGB** | 0,55 | 0,49 | 1,11 |  |  | U |  |
| **NUDT13** | -0,65 | -0,17 | 1,15 |  |  | U |  |
| **HIST2H3A** | -0,82 | -0,13 | 1,16 |  |  | U |  |
| **HNRNPH1** | -0,59 | -0,05 | 1,14 |  |  | U |  |
| **RPL11** | 0,10 | -0,70 | 1,23 |  |  | U |  |
| **PSMD13** | -0,88 | 0,34 | 1,23 |  |  | U |  |
| **TTC31** | -0,73 | 0,45 | 1,32 |  |  | U |  |
| **CDC123** | -0,40 | 0,27 | 1,37 |  |  | U |  |
| **KIF20B** | -0,73 | 0,82 | 1,58 |  |  | U |  |
| **RRBP1** | 0,17 | -0,33 | 2,32 |  |  | U |  |
| **RAB7A** | -0,80 | 0,67 | 3,36 |  |  | U |  |
