# Supplementary material for: Proteomic Analysis of Zn Depletion/Repletion in the Hormone-Secreting Thyroid Follicular Cell Line FRTL-5
Source: Nutrients. 2018 Dec 14;10(12):1981. doi: 10.3390/nu10121981 (PMC6315927; doi:10.3390/nu10121981)
Supplement: Supplementary file 1 [file nutrients-10-01981-s001.zip › Table S2.docx]

**Table S2:** Significantly enriched Biological Processes (Gene Ontology)

| TPEN vs CTRL | | | | | |
| --- | --- | --- | --- | --- | --- |
| Term | **Count** | **p value** | **Genes** | **UP Genes** | **DOWN genes** |
| GO:0005975~carbohydrate metabolic process | **6** | **0.000015** | **TPI1, TALDO1, SHPK, PYGB, MDH1, B4GALT5** | **TPI1, TALDO1, SHPK, PYGB, MDH1, B4GALT5** |  |
| GO:0002027~regulation of heart rate | **3** | **0.003876** | **DMD, CACNA1E, CALM1** | **CACNA1E, DMD** | **CALM1** |
| GO:0019682~glyceraldehyde-3-phosphate metabolic process | **2** | **0.005012** | **TPI1, TALDO1** | **TPI1, TALDO1** |  |
| GO:0009052~pentose-phosphate shunt, non-oxidative branch | **2** | **0.012485** | **TALDO1, SHPK** | **TALDO1, SHPK** |  |
| GO:0006412~translation | **5** | **0.014638** | **RRBP1, RPL9, RPL27A, RPL11, RPS5** | **RRBP1, RPL27A, RPL11, RPS5** | **RPL9** |
| GO:1901385~regulation of voltage-gated calcium channel activity | **2** | **0.019903** | **DMD, AHNAK** | **DMD** | **AHNAK** |
| GO:0060314~regulation of ryanodine-sensitive calcium-release channel activity | **2** | **0.029708** | **DMD, CALM1** | **DMD** | **CALM1** |
| GO:0006098~pentose-phosphate shunt | **2** | **0.032145** | **TPI1, TALDO1** | **TPI1, TALDO1** |  |
| GO:0043248~proteasome assembly | **2** | **0.034575** | **PSMD13, PSMD11** | **PSMD13** | **PSMD11** |
| GO:0010880~regulation of release of sequestered calcium ion into cytosol by sarcoplasmic reticulum | **2** | **0.049033** | **DMD, CALM1** | **DMD** | **CALM1** |
|  |  |  |  |  |  |
|  |  |  |  |  |  |
| RECOVERY vs TPEN | | | | | |
| Term | **Count** | **p value** | **Genes** | **UP Genes** | **DOWN genes** |
| GO:0006412~translation | **7** | **0.000850** | **RPL14, RPL27A, EIF5B, RPL5, RPL12, RPS5, RPS7** |  | **RPL14, RPL27A, EIF5B, RPL5, RPL12, RPS5, RPS7** |
| GO:0006364~rRNA processing | **4** | **0.001170** | **RPL14, WBP11, RPL5, RPS7** |  | **RPL14, WBP11, RPL5, RPS7** |
| GO:0098609~cell-cell adhesion | **4** | **0.024651** | **CNN3, RPL15, SPTBN1, AHNAK** | **AHNAK** | **CNN3, RPL15, SPTBN1** |
| GO:0007264~small GTPase mediated signal transduction | **4** | **0.032119** | **RAB8B, RAN, RAB6B, RAP1B** | **RAB6B, RAP1B** | **RAB8B, RAN** |
| GO:2000114~regulation of establishment of cell polarity | **2** | **0.046427** | **PTK2B, RAP1B** | **RAP1B** | **PTK2B** |
