# Supplementary material for: Proteomic Analysis of Zn Depletion/Repletion in the Hormone-Secreting Thyroid Follicular Cell Line FRTL-5
Source: Nutrients. 2018 Dec 14;10(12):1981. doi: 10.3390/nu10121981 (PMC6315927; doi:10.3390/nu10121981)
Supplement: Supplementary file 1 [file nutrients-10-01981-s001.zip › Table S3.docx]

**Table S3**: Significantly enriched molecular functions (Gene Ontology)

| TPEN vs CTRL | | | | | |
| --- | --- | --- | --- | --- | --- |
| Term | **Count** | **p value** | **Genes** | **UP Genes** | **DOWN genes** |
| GO:0005515~protein binding | **12** | **0.001877** | **RAB7A, APOB, TPI1, RAN, RRBP1, DMD, ERP29, ANXA5, RPS5, PPP1CB, HNRNPA1, CALM1** | **RAB7A, APOB, TPI1, RAN, RRBP1, DMD, ERP29, ANXA5, RPS5, PPP1CB** | **HNRNPA1, CALM1** |
| GO:0044822~poly(A) RNA binding | **10** | **0.002093** | **RAN, RRBP1, RPL15, RPL27A, SSB, RPL11, HNRNPH1, RPS5, HNRNPA1, AHNAK** | **RAN, RRBP1, RPL15, RPL27A, RPL11, HNRNPH1, RPS5** | **SSB, HNRNPA1, AHNAK** |
| GO:0019843~rRNA binding | **3** | **0.006542** | **RPL9, RPL11, RPS5** | **RPL11, RPS5** | **RPL9** |
| GO:0003735~structural constituent of ribosome | **5** | **0.022882** | **RPL9, RPL15, RPL27A, RPL11, RPS5** | **RPL15, RPL27A, RPL11, RPS5** | **RPL9** |
| GO:0003723~RNA binding | **5** | **0.031443** | **RPL9, SSB, HNRNPH1, RPS5, HNRNPA1** | **HNRNPH1, RPS5** | **RPL9, SSB, HNRNPA1** |
| GO:0019904~protein domain specific binding | **4** | **0.045295** | **ST13, RAN, SKIL, CALM1** | **RAN, SKIL** | **ST13, CALM1** |
|  |  |  |  |  |  |
|  |  |  |  |  |  |
| RECOVERY vs TPEN | | | | | |
| Term | **Count** | **p value** | **Genes** | **UP Genes** | **DOWN genes** |
| GO:0044822~poly(A) RNA binding | **14** | **0.000033** | **RPL14, RAN, RPL15, RPL27A, EIF5B, WBP11, SSB, RPS5, RPS7, SFPQ, SPTBN1, RPL5, RPL12, AHNAK** | **SSB, SFPQ, AHNAK** | **RPL14, RAN, RPL15, RPL27A, EIF5B, WBP11, RPS5, RPS7, SPTBN1, RPL5, RPL12** |
| GO:0032403~protein complex binding | **8** | **0.000141** | **ST13, GNB1, RAN, PTK2B, GNA11, SPTBN1, ATP5O, RAP1B** | **ST13, RAP1B** | **GNB1, RAN, PTK2B, GNA11, SPTBN1, ATP5O** |
| GO:0003735~structural constituent of ribosome | **7** | **0.001921** | **RPL14, RPL15, RPL27A, RPL5, RPL12, RPS5, RPS7** |  | **RPL14, RPL15, RPL27A, RPL5, RPL12, RPS5, RPS7** |
| GO:0003924~GTPase activity | **5** | **0.003364** | **GNB1, RAN, GNA11, EIF5B, RAP1B** | **RAP1B** | **GNB1, RAN, GNA11, EIF5B** |
| GO:0098641~cadherin binding involved in cell-cell adhesion | **5** | **0.004891** | **CNN3, RPL15, SPTBN1, AHNAK, VCL** | **AHNAK** | **CNN3, RPL15, SPTBN1, VCL** |
| GO:0005525~GTP binding | **6** | **0.006506** | **RAB8B, RAN, GNA11, EIF5B, RAB6B, RAP1B** | **RAB6B, RAP1B** | **RAB8B, RAN, GNA11, EIF5B** |
| GO:0004871~signal transducer activity | **4** | **0.011891** | **GNB1, PTK2B, GNA11, VOPP1** | **VOPP1** | **GNB1, PTK2B, GNA11** |
| GO:0019003~GDP binding | **3** | **0.015085** | **RAB8B, RAN, RAP1B** | **RAP1B** | **RAB8B, RAN** |
| GO:0047391~alkylglycerophosphoethanolamine phosphodiesterase activity | **2** | **0.015520** | **GNB1, GNA11** |  | **GNB1, GNA11** |
