# Supplementary figures and images for: Proteomic Analysis of Zn Depletion/Repletion in the Hormone-Secreting Thyroid Follicular Cell Line FRTL-5
Source: Nutrients. 2018 Dec 14;10(12):1981. doi: 10.3390/nu10121981 (PMC6315927; doi:10.3390/nu10121981)

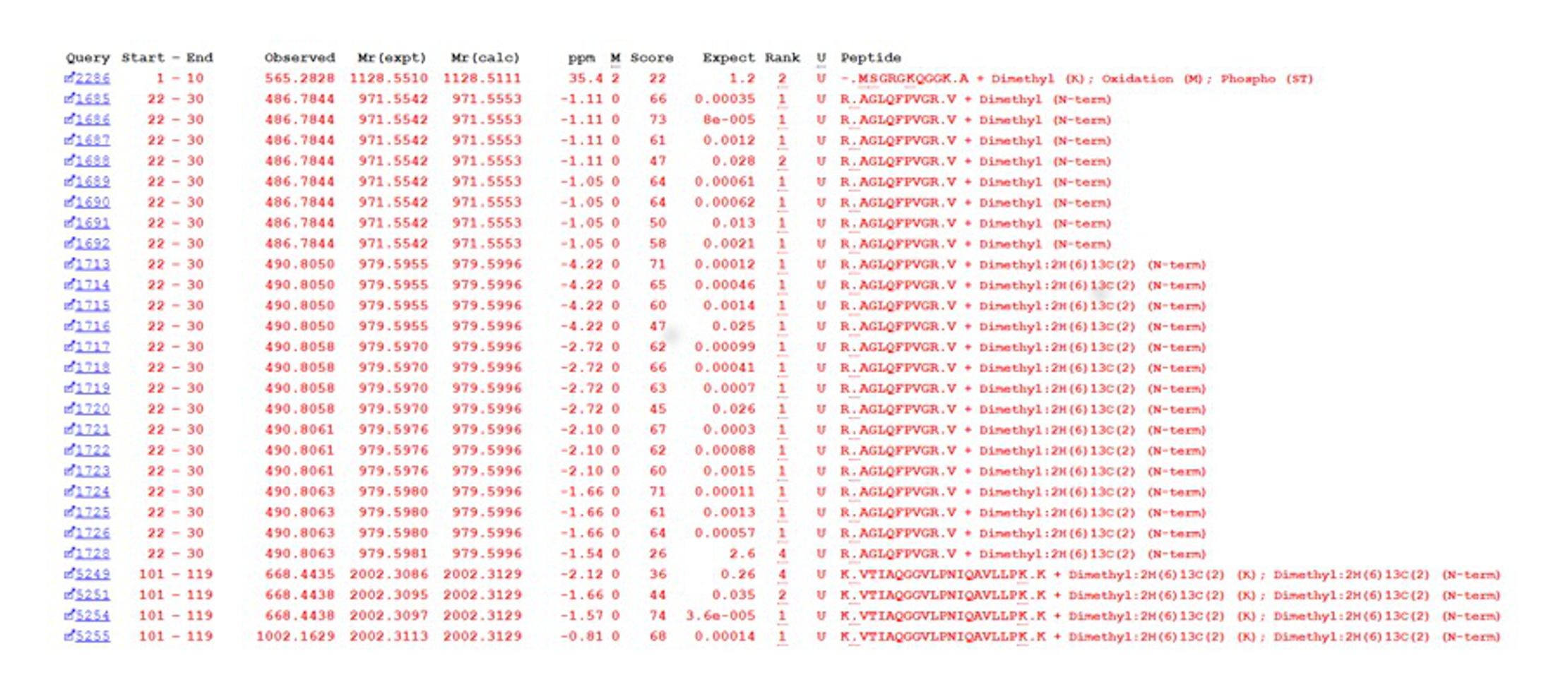

Supplement: Supplementary file 1 [file nutrients-10-01981-s001.zip › Figure S1.tif]

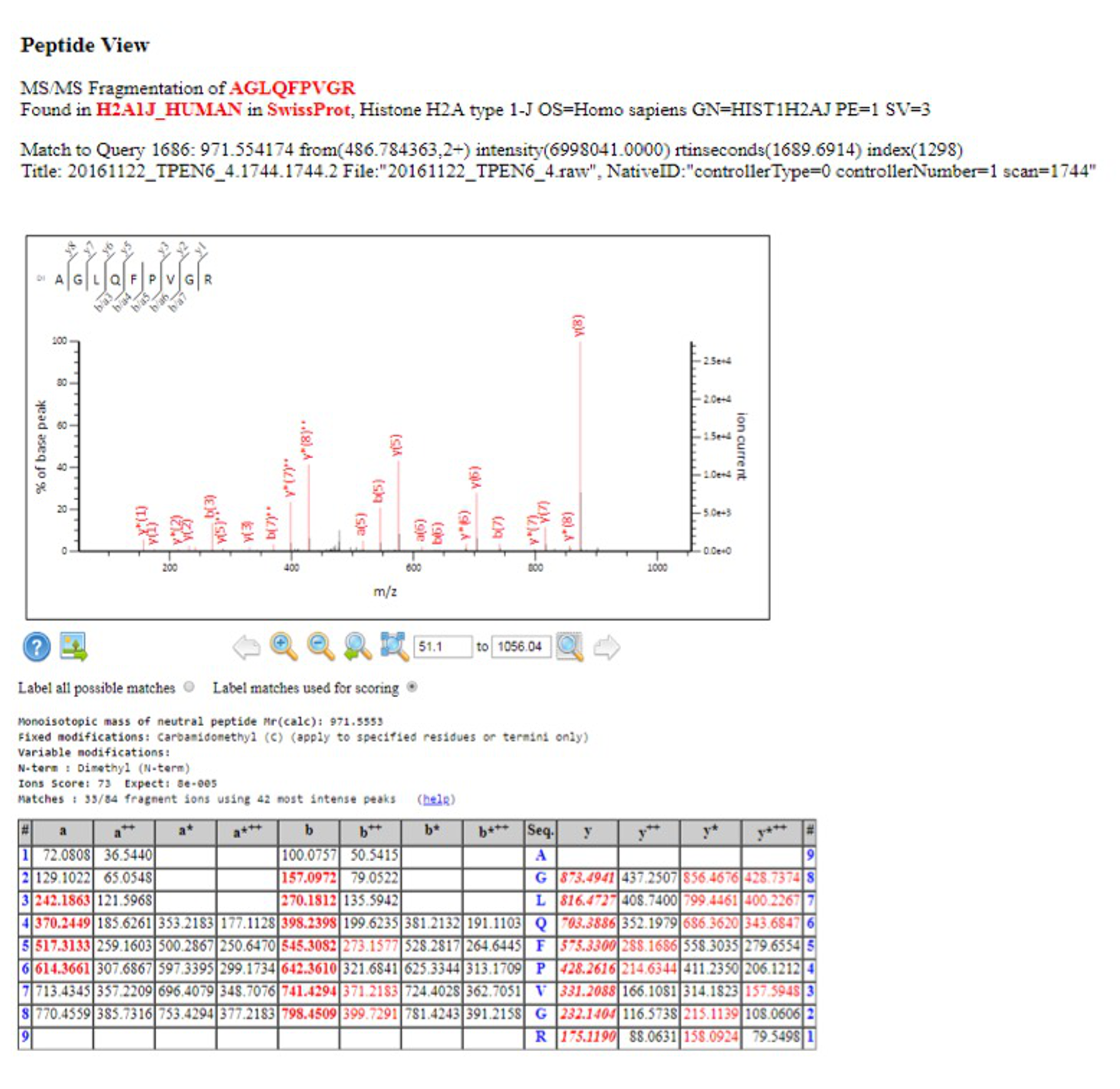

Supplement: Supplementary file 1 [file nutrients-10-01981-s001.zip › Figure S2.tif]

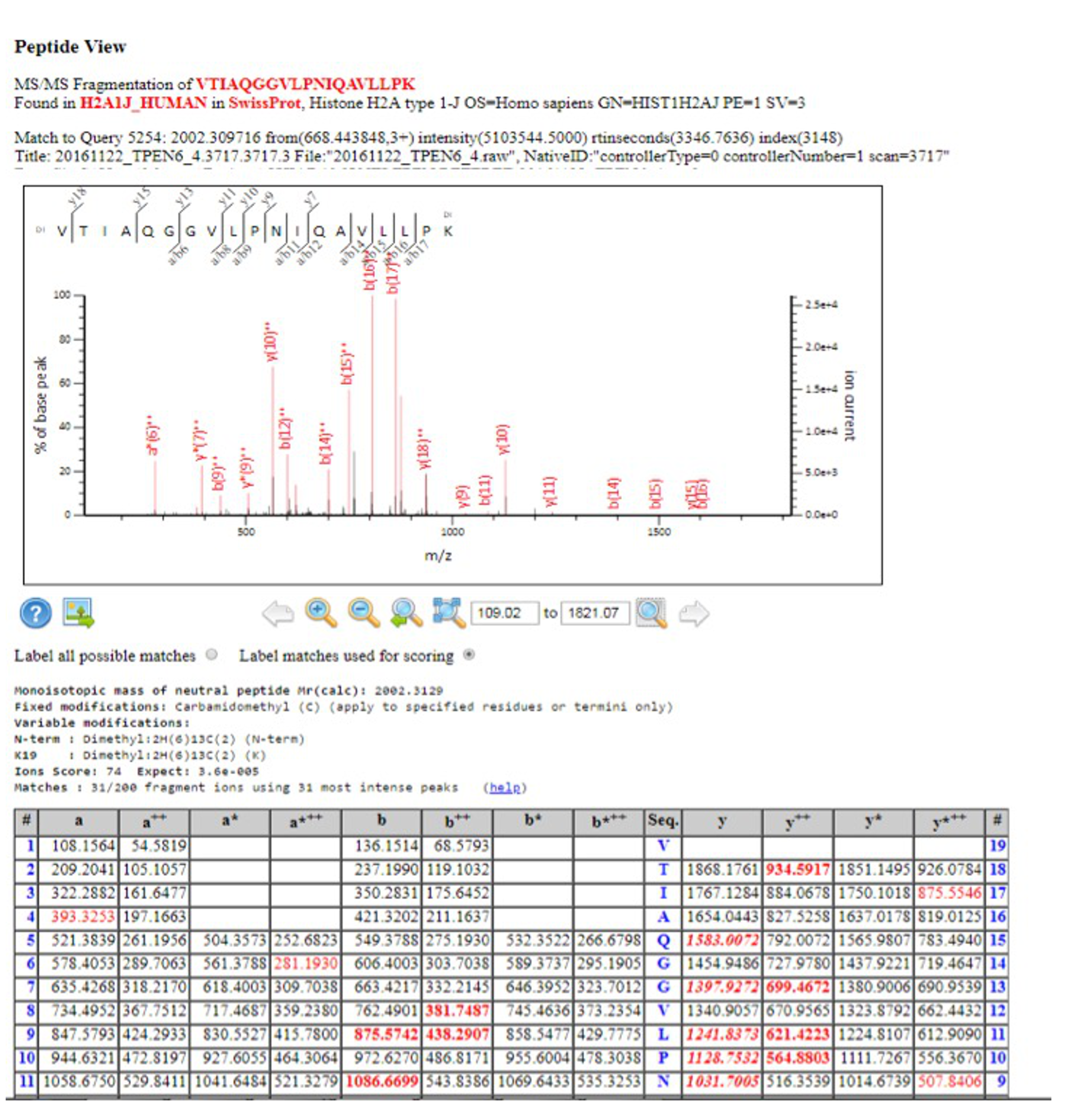

Supplement: Supplementary file 1 [file nutrients-10-01981-s001.zip › Figure S3.tif]
